# Amygdala and Insula Retraining (AIR) Significantly Reduces Fatigue and Increases Energy in People with Long COVID

**DOI:** 10.1155/2023/7068326

**Published:** 2023-07-17

**Authors:** Loren L. Toussaint, Alexandra J. Bratty

**Affiliations:** ^1^Department of Psychology, Luther College, 700 College Drive, Decorah, IA 52101, USA; ^2^AB Research Consulting, 508 Summer Mesa Drive, Las Vegas, NV 89148, USA

## Abstract

Long COVID affects approximately 10–30% of individuals after an acute COVID-19 infection (Ceban, Ling, et al. 2022; Ortona and Malorni, 2022). Numerous symptoms, including extreme fatigue, can persist for months, resulting in social and economic hardship for individuals and their families (Ortona and Malorni 2022). Therefore, approaches that offer some relief from Long COVID are urgently needed. Research suggests that Long COVID symptoms are akin to those of chronic conditions, such as myalgic encephalomyelitis/chronic fatigue syndrome (ME/CFS) and are likely caused by inflammation and immune dysfunction (Scordo et al., 2021). Amygdala and Insula Retraining (AIR), a neuroplasticity program, has successfully alleviated chronic conditions (Gupta 2010; Sanabria-Mazo et al. 2020; Toussaint et al. 2012). In this randomized controlled trial, AIR was tested against a structurally equivalent health and wellness intervention for its effectiveness in treating the symptom of fatigue among Long COVID sufferers. Results showed a significant decrease in participants' fatigue and a significant increase in their energy after the 3-month AIR intervention. Additionally, the AIR group experienced more significant outcomes than the active control group. The AIR group demonstrated a fatigue reduction effect size four times that of the active control group, and the absolute reduction in mean scores for the AIR group was more than double that of the control group. Furthermore, the AIR group showed an effect size in energy enhancement twice that of the active control group, and the absolute increase in energy mean scores for the AIR group was almost double that of the control group. These novel findings suggest AIR is a viable means of reducing fatigue and increasing energy among Long COVID patients. Limitations and future research are discussed.

## 1. Introduction

Long COVID refers to a range of 200 possible symptoms that can persist for several months [1] for roughly 10–30% of individuals after their acute COVID-19 infection has been resolved [1, 2]. Symptoms include extreme fatigue, myalgia, and cognitive impairment [3], and effective means of coping with Long COVID are urgently needed. Pharmaceutical, biological, dietary, homeopathic, and rehabilitative remedies have been tested, yet help for Long COVID remains elusive [4].

Long COVID symptoms are likely caused by chronic inflammation and immunological dysregulation in the body [1]. They are similar to those of myalgic encephalomyelitis/chronic fatigue syndrome (ME/CFS), a chronic disease characterized by immune and cognitive dysfunction [5]. Therefore, it is possible that treatments for ME/CFS could be effective in resolving Long COVID [5]. An amygdala and insula retraining (AIR) intervention, known as The Gupta Program, has been successful in alleviating symptoms of ME/CFS [6, 7] and other chronic conditions, like fibromyalgia [7, 8]. Thus, the current study aimed to test the effectiveness of the AIR intervention for Long COVID.

The AIR intervention is hypothesized to strengthen neurological inhibitory mechanisms in the prefrontal cortex, insula, amygdala, and anterior and posterior cingulate. This process helps reduce the magnification of incoming bodily signals and the hyper-stimulation of the autonomic nervous and immune systems by the amygdala and the insula, thereby allowing the systems to return to a normal state of balance [6]. The AIR intervention primarily involves specialized neuroplasticity techniques to retrain the immune and nervous systems' hyperactivity.

The study aimed to test the AIR program's effectiveness in reducing prolonged symptoms of fatigue following COVID-19 infection. Additionally, it aimed to evaluate the effectiveness of AIR compared to a general health intervention. To our knowledge, no research has yet examined the impact of a neuroplasticity program in remedying Long COVID symptoms. Consequently, this novel study attempted to generate potential insights that could inform future treatment. The following hypotheses were explored:

(H1) There will be a significant decrease in participants' fatigue after the AIR intervention
(H2) Compared to an active control group, the experimental group will experience a significant decrease in participants' fatigue after the AIR intervention

## 2. Materials and Methods

### 2.1. Participants

The study had an experimental design with four measurement points conducted among *N* = 100 individuals. The inclusion criteria required that participants be of ages 21–65 and suffering from postviral symptoms at least 3 months after an acute COVID-19 infection. Exclusion criteria included those who reported suffering from the following conditions to avoid any confounds that could be caused by additional ailments: hypo and hyperthyroidism (untreated), adrenal disorders (untreated), diabetes, multiple sclerosis, hepatitis, cancer (active), depression (untreated), chronic steroid use, acute inflammatory rheumatological conditions, obstructive sleep apnea, and narcolepsy. The study was conducted according to the guidelines of the Declaration of Helsinki and approved by the Institutional Review Board (or Ethics Committee) of Luther College (protocol code 30.20 and October 22, 2020).

Participants were recruited from three English-language Facebook groups: Long Haul COVID Fighters (80+ Days), Long Haul COVID Fighters (30+ Days), and Long COVID Support Group. The sample comprised *n* = 86 women (86%), *n* = 12 men (12%), *n* = 1 nonbinary, and *n* = 1 who declined to answer. The mean age was 43.6 years. One-fifth (*n* = 20) had less than a college education, *n* = 43 had a bachelor's degree, *n* = 32 had a master's degree, and *n* = 5 held a doctorate. Half of the participants (*n* = 50) were randomly assigned to the AIR intervention and the other half (*n* = 50) to the active control group using an Excel function that generated a list of random assignments following enrollment in the study.

A series of chi-square tests and an independent *t*-test indicated that the AIR intervention and active control groups did not differ significantly on any of the demographic variables: gender [*χ*^2^ (2) = 0.99, *p*=0.610], age [*t* (98) = −0.053, *p*=0.957 (two-tailed)], and education [*χ*^2^ (7) = 8.70, *p*=0.275]. Additionally, a series of chi-square tests and an independent *t*-test were performed to compare the demographic variables of participants who completed the full 3-month study (*n* = 40) versus those who dropped out at some point (*n* = 60). No significant difference was found between groups for gender [*χ*^2^ (2) = 4.00, *p*=0.136] and education [*χ*^2^ (7) = 6.52, *p*=0.481]. However, there was a significant difference in age across the two groups [*t* (98) = −2.46, *p*=0.016 (two-tailed)]. The mean age of those who dropped out was 41.6, while the mean age of those who completed the study was 46.3.

### 2.2. Procedure

Participants were randomly assigned to the AIR intervention or an active control group. The AIR intervention group attended an online workshop to introduce participants to 6+---------------------------the educational neuroplasticity program of Amygdala and Insula Retraining (AIR) and received supporting materials online and by mail. The main AIR practices include specialized neuroplasticity techniques. The primary process is designed to interrupt adverse somatic signals and mental patterns and direct individuals to create new, positive neural pathways that indicate safety to the brain through repetition. Secondary activities support the neuroplasticity techniques. These include mindfulness-based meditation, where individuals focus on the present; alternate nostril breathing, where individuals hold one nostril shut while breathing through the other, then change nostrils and repeat; and other lifestyle therapies, such as suggested general health supplements and cultivating a calming morning ritual that eases people into their day.

Participants were asked to practice the AIR intervention for 40–60 minutes daily, including the main neuroplasticity processes, a few minutes of alternate nostril breathing, and a simple 20-minute mindfulness meditation practice. In addition, throughout the day, they were asked to practice abbreviated versions of the neuroplasticity techniques to interrupt somatic signals further and retrain the Amygdala and Insula's hypothesized responses. These retraining techniques took about 30–60 seconds to enact each time. The participants received weekly webinars to support their training and the opportunity to ask questions within the webinars. Finally, participants had timely access to the study investigators for any immediate questions or challenges that arose with the practice and were offered optional online support with a coach trained in AIR.

The active control group received an online educational program for general health and well-being (12 weeks to wellness). It involved general advice on diet, exercise, energy, nutrition, sleep, and other lifestyle interventions. The group was encouraged to live an overall healthier lifestyle and to put the knowledge gained from the program into practice. Participants in this program learned about eating healthy foods, managing stress, and developing an active work-life balance. In addition to making behavioral changes, the program emphasized the importance of individuals shifting their internal attitudes and beliefs about wellness. Participants attended weekly webinars, were provided with online resources, and were offered optional online support with a coach trained in the 12 weeks to wellness program.

**Figure 1:**
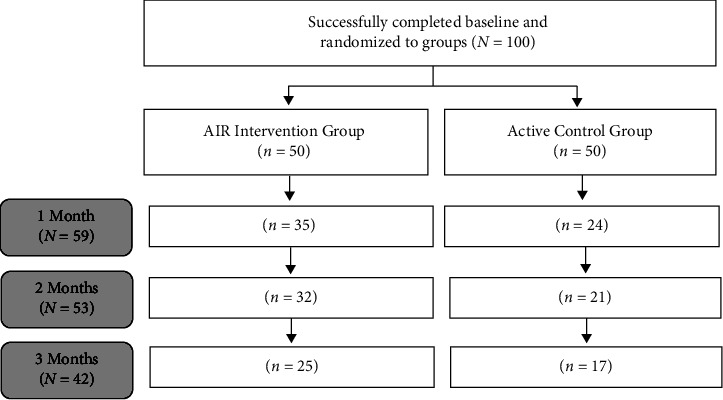
Flowchart of participants. *Note*. AIR = Amygdala and Insula Retraining. *n* = 2 participants in the AIR group did not complete measures at all four time intervals.

The AIR and active control interventions were free for participants, and no incentives were used to encourage participation. The control group was also offered complimentary access to AIR after the 3-month study. There were four points of measurement: baseline (before the intervention), 1 month, 2 months, and 3 months (upon completion of the intervention).  Figure 1 displays the flowchart of participants and sample sizes for the groups at each point during the study. The final number of participants in month 3 was *N* = 42. However, two respondents did not complete the measures at all four time intervals. Therefore, the overall retention rate for full completion of the study (i.e., participants completed all four measures), from baseline (*N* = 100) to month 3 (*N* = 40), was 40%. This retention rate for an online intervention is higher than typically observed [9]. Notably, the retention rate for the AIR group was higher (*n* = 23; 46%) than that for the control group (*n* = 17; 34%).

### 2.3. Measures

#### 2.3.1. Multidimensional Fatigue Inventory

Fatigue was measured using the multidimensional fatigue inventory (MFI) [10]. It is a 20-item scale that assesses five dimensions of fatigue (four items per dimension): general fatigue, physical fatigue, reduced motivation, reduced activity, and mental fatigue. The MFI scale developers recommended using the general fatigue subscale as a global fatigue index because of its sensitivity to changes in fatigue level [10, 11]. Participants respond to each item using a 5-point Likert-type scale that ranges from the lowest score of 1, which represents a response of *yes that is true*, to the highest score of 5, which represents a response of *no that is not true*. Higher total scores indicate greater levels of fatigue. The developers of the scale reported internal reliability ranging from *α* = 0.53 to *α* = 0.93 [10]. In the present study, the internal reliability score for general fatigue was acceptable across most points of measurement (baseline: *α* = 0.54; month 1: *α* = 0.72; month 2: *α* = 0.90; month 3: *α* = 0.86).

#### 2.3.2. Short Form Health Survey (SF-36)

The SF-36 questionnaire [12–14] measured several quality-of-life factors. The 36-item scale assesses eight dimensions (ranging from two to 10 items per dimension): physical functioning, role limitations due to physical health, role limitations due to emotional problems, energy, emotional well-being, social functioning, pain, and general health. The energy subscale was of most interest in the present study. Items include binary yes/no responses and some Likert-type scales that vary from lowest scores representing responses such as *poor*, *much worse*, and *limited a lot* and highest scores representing responses such as *excellent, much better*, *and not limited at all*. Higher total scores indicate a more favorable health status. Validation of the scale reported internal reliability of *α* = 0.85 [15]. In the present study, the internal reliability score for energy was acceptable across all measurement points (baseline: *α* = 0.73; month 1: *α* = 0.87; month 2: *α* = 0.90; month 3: *α* = 0.91).

### 2.4. Analyses

A series of two-way mixed ANOVAs were used to analyze the data in IBM SPSS v26.0. Given the study's emphasis on reducing symptoms of fatigue, the analysis focused only on the fatigue-related dimensions of each scale. As noted above, *N* = 100 participants completed the baseline measure (*n* = 50 AIR; *n* = 50 control), but only *N* = 40 completed all four measures (*n* = 23 AIR; *n* = 17 control). Therefore, expectation maximization, last-observation-carried-forward, and listwise methods for missing data were used to ensure unbiased statistical estimates.

The data were normal, with skewness and kurtosis scores within the ±2 acceptable ranges. Other assumptions required for a two-way mixed ANOVA were met, except for sphericity, which was violated for both subscales. Therefore, the Greenhouse-Geisser correction was used in reporting. Additionally, boxplots indicated three outliers in the MFI general fatigue data and one outlier in the SF-36 energy data. However, none of the outliers were extreme or considered aberrant in the data set. Therefore, all outliers were retained for analysis.

When running the two-way mixed ANOVAs, tests of within-subjects were consulted first to establish if there was a significant interaction between time and group. Subsequently, differences between groups at each time interval were examined, as well as changes within each group over time. The Bonferroni correction was used for pairwise comparisons, except where otherwise noted.

## 3. Results

### 3.1. General Fatigue (MFI Scale)

Analysis of the general fatigue subscale from the MFI scale using expectation maximization (EM) methods revealed that the groups changed significantly over time with a medium effect size: *F* (1.95, 191.10) = 7.04, *p*=0.001, partial *η*^2^ = 0.067. As shown in Table 1, there was no significant difference in general fatigue between the groups at baseline. However, the AIR group posted lower fatigue scores than the control group for months 1–3. The difference between the AIR and active control groups at month 1 was significant with a medium effect size. It was also significant at month 2 with a small effect size and approaching a significant difference at month 3 with a small effect size. Thus, general fatigue mean scores were significantly lower for the AIR group than the active control group at months 1 and 2 and approached a significantly lower mean score at month 3.

Notably, as shown in Table 1, the total decrease in mean scores for general fatigue from baseline to month 3 for the AIR group (3.38) was more than double that of the active control group (1.40). Moreover, research validating the MFI scale found the mean score for the general fatigue subscale among US adults was 12.90 [16]. Therefore, participants using AIR more readily approached normal levels of general fatigue by month 3.

Over time, results for the active control group showed a significant reduction in general fatigue with a medium effect size, *F* (1.95, 95.54) = 4.72, *p*=0.012, and partial *η*^2^ = 0.088. Pairwise comparisons showed that this significant reduction in mean fatigue scores occurred between baseline and month 3 (*p*=0.037), indicating that fatigue significantly declined for active control group participants by the end of their 12-week wellness program.

Over time, results for the AIR group were also significant and to a greater extent than those of the active control group. The AIR group demonstrated a very large effect size that was more than four times the control group effect size: *F* (1.93, 94.43) = 33.85, *p* < 0.001, partial *η*^2^ = 0.409. Pairwise comparisons showed a significant reduction in mean fatigue scores occurring between baseline and all other time points (*p* < 0.001), indicating that compared to the baseline, fatigue was significantly reduced at every time point (months 1, 2, and 3) for AIR participants.

**Figure 2:**
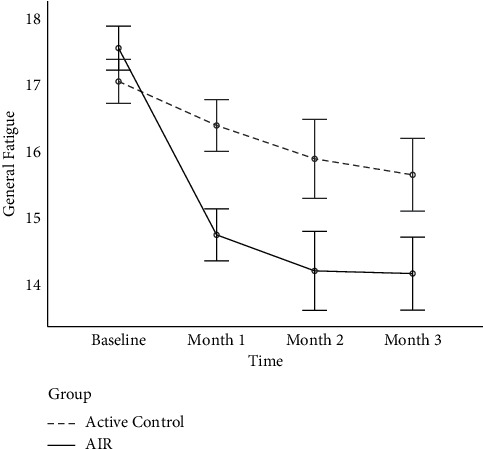
Average levels of general fatigue for active control and AIR groups across the four study timepoints. *Note*. AIR = Amygdala and Insula Retraining. Error bars represent standard error.

Figure 2 shows the estimated marginal means plot for general fatigue across time and group. Though both groups posted reduced fatigue scores at each time interval, it is notable that the AIR group experienced a marked decline in just one month compared to the active control group.

The same analyses were performed to verify findings, using the last observed value carried forward (LOCF) method for missing data (*N* = 100) and among only the respondents who completed all the study measures at each time interval (i.e., *N* = 40). Both approaches returned nearly identical results. The LOCF analysis indicated the groups changed significantly over time with a small effect size, *F* (1.81, 177.41) = 5.24, *p*=0.008, and partial *η*^2^ = 0.051. There was no significant difference between groups at any time interval (baseline: *p*=0.286, month 1: *p*=0.116, month 2: *p*=0.167, and month 3: *p*=0.115). However, both groups experienced a significant decline in fatigue over time, with the AIR group posting a more significant result and a larger effect size. Active control: *F* (1.91, 93.73) = 4.37, *p*=0.017, and partial *η*^2^ = 0.082. AIR group: *F* (1.72, 84.48) = 17.15, *p* < 0.001, and partial *η*^2^ = 0.259. Pairwise comparisons indicated the active control group had a significant decrease in fatigue between baseline and month 3 (*p*=0.040). In contrast, the AIR group experienced a significant decrease between the baseline and all time points (*p* < 0.001).

The analysis among the 40 respondents who completed all the study measures at each interval indicated that the change in groups over time approached statistical significance, *F* (1.93, 73.48) = 3.10, *p*=0.053, and partial *η*^2^ = 0.075. There was no significant difference between groups at any time interval (baseline: *p*=0.703, month 1: *p*=0.117, month 2: *p*=0.132, and month 3: *p*=0.146). Over time, the change in fatigue in the active control group was nonsignificant, *F* (1.97, 31.52) = 2.37, *p*=0.111, and partial *η*^2^ = 0.129. However, the AIR group experienced a significant decrease in fatigue over time with a very large effect size, *F* (1.74, 38.37) = 18.59, *p* < 0.001, and partial *η*^2^ = 0.458. Pairwise comparisons showed that the AIR group experienced a significant decrease between the baseline and all time points (*p* < 0.001). Thus, these additional analyses provided further evidence that AIR group participants benefited from a greater reduction in fatigue than the active control group over time.

### 3.2. Energy/Fatigue (SF-36 Scale)

Analysis for the energy subscale from the SF-36 scale using expectation maximization (EM) methods revealed that the change in groups over time approached statistical significance: *F* (2.41, 236.09) = 2.38, *p*=0.084, and partial *η*^2^ = 0.024. However, no significant difference was found between groups at any time interval, as shown in Table 2.

Results for both groups over time indicated a significant difference in energy scores: *F* (2.41, 236.09) = 31.26, *p* < 0.001, partial *η*^2^ = 0.242. The active control group demonstrated a significant increase in energy with a large effect size, *F* (2.44, 119.57) = 9.63, *p* < 0.001, partial *η*^2^ = 0.164. Pairwise comparisons showed a significant increase in energy scores between baseline and month 1 (*p* < 0.001), baseline and month 2 (*p*=0.004), and baseline and month 3 (*p*=0.002), suggesting that compared to the baseline, energy significantly increased at every time point (months 1, 2, and 3) for active control participants.

The AIR group also experienced a significant increase in energy over time, with a large effect size double that of the active control group: *F* (2.22, 108.79) = 24.23, *p* < 0.001, partial *η*^2^ = 0.331. Pairwise comparisons indicated a significant increase in energy between baseline and all other time points (*p* < 0.001), suggesting that AIR group participants experienced an increase in energy at all intervals compared to the baseline. In addition, AIR participants experienced a significant increase in energy between months 1 and 3 (*p*=0.007) and approached a significant increase between months 1 and 2 (*p*=0.055).

**Figure 3:**
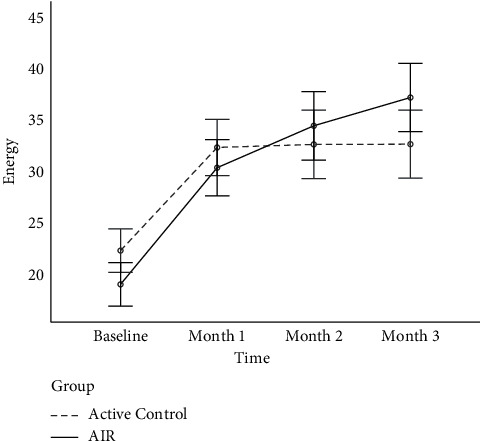
Average levels of energy for active control and AIR groups across the four study timepoints. *Note*. AIR = Amygdala and Insula Retraining. Error bars represent standard error.

Figure 3 shows the estimated marginal means plot for energy across time and group. Though the difference between groups was nonsignificant at all time points, it is worth noting that the AIR group started at a marginally lower mean score at baseline and climbed to a higher final energy level at month 3. Indeed, as shown in Table 2, the absolute increase in mean scores for energy for the AIR group (18.26) was almost twice that of the active control group (10.40).

The same analyses were performed using the LOCF method for missing data (*N* = 100) and among only the respondents who completed all the study measures at each time interval (i.e., *N* = 40). The results for those who completed all measures were virtually identical to the EM imputation method findings. However, using the LOCF method yielded more significant findings. The groups changed significantly over time with a small effect size: *F* (2.10, 205.48) = 3.65, *p*=0.026, partial *η*^2^ = 0.036. Still, there were no significant differences between groups at any time interval (baseline: *p*=0.274, month 1: *p*=0.613, month 2: *p*=0.305, month 3: *p*=0.452).

Results for the active control group over time showed a significant increase in energy with a medium effect size: *F* (1.93, 94.45) = 4.26, *p*=0.018, and partial *η*^2^ = 0.080. Pairwise comparisons using the Bonferroni correction showed no significant result between time points, perhaps because of the overly conservative nature of the method [17]. However, with the Least Significant Difference (LSD) adjustment, there were significant increases between the baseline and all other points (month 1: *p*=0.020, month 2: *p*=0.021, and month 3: *p*=0.020), suggesting that control group participants' energy improved across time. Results for the AIR group over time were more significant with a large effect size: *F* (2.20, 107.64) = 16.90, *p* < 0.001, and partial *η*^2^ = 0.256. Pairwise comparisons showed a significant increase in mean energy scores between baseline and all other time points (*p* < 0.001), with both the Bonferroni and LSD adjustments, indicating that energy was significantly enhanced at month 1, month 2, and month 3 for AIR participants.

## 4. Discussion

The aim of this study was twofold. First, to evaluate the AIR intervention's impact in reducing prolonged symptoms of fatigue from Long COVID. Second, to test the effectiveness of AIR compared to a structurally equivalent general health and wellness intervention. Results supported both hypotheses. There was a significant decrease in participants' fatigue after the 3-month AIR intervention, and compared to an active control group, the AIR group experienced a more significant decrease in participants' fatigue. For general fatigue, the effect size of the reduction in the AIR group was four times that of the active control group. Additionally, the absolute decrease in fatigue mean scores for the AIR group was more than double that experienced by the control group. Furthermore, the final general fatigue mean score for the AIR group approached standardized healthy norms [16]. Similarly, the AIR group also demonstrated a significant increase in energy over time, with a large effect size that was double that of the active control group, and the absolute increase in energy mean scores for the AIR group was almost double that of the active control group. These findings provide initial evidence that AIR is a viable and effective approach to mitigating fatigue from Long COVID.

The results may be explained by the AIR intervention hypothesis, namely, that the sympathetic nervous and immune systems are triggered into an overactive and hypervigilant state following a physical illness or stressor, such as COVID-19. This overactivity of the nervous and immune systems manifests as various bodily symptoms, such as prolonged fatigue [6, 18]. The AIR intervention employs neuroplasticity techniques designed to create new neural pathways and retrain the amygdala and insula so the immune and autonomic nervous systems can return to homeostasis. Indeed, other research has supported this theory of immune conditioning in the insula [19], reinforcing the concept of using neural retraining to reduce stimulation in the nervous and immune systems.

Finally, results from the present study support the hypothesis that interventions successful in treating chronic conditions, such as ME/CFS, might be transferrable to addressing Long COVID symptoms because such ailments are likely caused by inflammation and immune dysfunction in the body [1, 5]. Certainly, the current results are consistent with prior research that demonstrated the effectiveness of AIR in alleviating chronic conditions. For example, in combination with treatment-as-usual, AIR effectively reduced symptoms of fibromyalgia, including pain, fatigue, and depressive symptoms [8]. Similarly, another study found that AIR helped address ME/CFS symptoms such as energy, pain, and fatigue [7]. Thus, the effectiveness of AIR in alleviating chronic conditions seems to extend at least to reducing the symptom of fatigue in Long COVID.

### 4.1. Strengths and Limitations

This study had several strengths. The primary strength is the novelty of this research. To our knowledge, this is the first study demonstrating the effectiveness of a neuroplasticity program (AIR) in reducing the Long COVID symptom of prolonged fatigue. Second, an experimental research design was used to identify cause-and-effect outcomes. Finally, the research design included an active control group rather than a wait-list control group. Thus, the AIR intervention was tested against a structurally equivalent intervention intended to improve health and wellness.

However, the study was not without various limitations. These included a convenience sample, a relatively small sample size that may have reduced statistical power, self-reported measures, and a study design that may have impacted results. The sample was recruited from Long COVID support groups on social media, which relied on participants' self-report of Long COVID symptoms for 3 months or more. Thus, it is possible some participants believed they had Long COVID when they did not. However, the study was conducted in the first half of 2021, when the health community's diagnosis and understanding of Long COVID were nascent. Therefore, by necessity, recruitment heavily depended on participant self-diagnosis, and it is unclear if a more stringent verification method could have been used in the circumstances. Moreover, participants were not asked if they used other therapies for their Long COVID condition. Instead, the exclusion criteria served as a proxy to eliminate the possibility of including participants with comorbidities, taking prescription drugs, or engaging in other treatments. Still, any potential confounds created by these limitations of diagnosis or additional therapies would have been mitigated by randomizing participants to the AIR or active control groups and would not have prevented the detection of the intervention impact.

The study design also presented a limitation by only including an active control group and not a passive control group. The active control group provided a robust test for the effectiveness of AIR. However, with no passive control group, it is impossible to know how much more effective AIR would be compared to Long COVID symptoms following their natural course over 3 months. Furthermore, the study design did not allow for a follow-up measure to assess the longer-term impacts of AIR or the active control treatment on the symptom of fatigue. Finally, the study relied on self-reported measures rather than observations by health professionals. Arguably, the evaluation of fatigue is best reported by the self. Still, including objective biomarkers, such as oxidative stress and antioxidative activity, could have enhanced the investigation.

Future research should address these limitations and explore new areas of investigation. Forthcoming studies should recruit participants from well-established Long COVID clinics, incorporate objective biomarkers and observations from health professionals, and include a waiting control condition. Other suggestions include measuring additional symptoms of Long COVID and conducting larger studies to further knowledge of AIR as an approach to easing fatigue in people living with Long COVID.

## 5. Conclusion

This novel study was the first to demonstrate the effectiveness of a neuroplasticity program (AIR) in addressing the Long COVID symptom of prolonged fatigue. Participants who received the AIR intervention experienced a significant reduction in fatigue and an increase in energy. Moreover, the AIR intervention yielded more significant fatigue reduction and energy enhancement results than an active control group. The effect size of AIR's impact on fatigue was four times greater than that of the active control group, and the absolute reduction in fatigue scores for the AIR cohort was more than twice that of the control. Similarly, the effect size of AIR's impact on energy was double that of the active control group, and the absolute increase in energy scores for the AIR group was almost twice that of the control group. These findings are both timely and pertinent, as so little is known about how to treat Long COVID and so many patients suffer from it after the acute infection of COVID-19. The present research suggests that AIR—a low-cost and widely available intervention—could help alleviate the common symptom of prolonged fatigue in Long COVID.

## Figures and Tables

**Table 1:** Mean scores for general fatigue (MFI).

General fatigue (MFI)	Groups		Difference	*F* (1, 98)	*P*	*η* ^2^
	AIR (*n* = 50)	Control (*n* = 50)				
	*M* (SE)	*M* (SE)				
Baseline	17.54 (0.330)	17.04 (0.330)	0.05	1.15	0.286	0.012
Month 1	14.74 (0.388)	16.38 (0.388)	1.64	8.91	0.004	0.083
Month 2	14.20 (0.592)	15.88 (0.592)	1.68	4.03	0.048	0.039
Month 3	14.16 (0.546)	15.64 (0.546)	1.48	3.67	0.058	0.036
Total decrease	−3.38	−1.40				
Percent decrease	19.27%	8.22%				

*Note*. AIR = Amygdala and Insula Retraining; difference = difference in mean scores between AIR and control; total decrease = difference between baseline and month 3. Data are shown for the EM imputation method.

**Table 2:** Mean scores for energy (SF-36).

Energy (SF-36)	Groups		Difference	*F* (1, 98)	*P*	*η* ^2^
	AIR (*n* = 50)	Control (*n* = 50)				
	*M* (SE)	*M* (SE)				
Baseline	18.90 (2.122)	22.20 (2.122)	3.30	1.21	0.274	0.012
Month 1	30.30 (2.748)	32.28 (2.748)	1.98	0.26	0.612	0.003
Month 2	34.40 (3.353)	32.58 (3.353)	1.82	0.15	0.702	0.002
Month 3	37.16 (3.329)	32.60 (3.329)	4.56	0.94	0.335	0.009
Total increase	18.26	10.40				
Percent increase	96.61%	46.85%				

*Note*. AIR = Amygdala and Insula Retraining. Difference = difference in mean scores between AIR and control. Total increase = difference between baseline and month 3. Data shown for the EM imputation method.

## Data Availability

The data are not publicly available because the sample consists of vulnerable adults with a chronic medical condition. Individuals did not agree in the consenting process to have their data shared and expect their data to be kept strictly confidential.
